# Redo aortic valve replacement after partial upper sternotomy (hemisternotomy) in a patient with idiopathic thrombocytopenia: a case report

**DOI:** 10.1186/1757-1626-1-422

**Published:** 2008-12-29

**Authors:** Hiba Fatayer, Steven Griffin, Aristotle D Protopapas

**Affiliations:** 1 East Yorkshire Cardiothoracic Surgical Centre, Castle Hill, Kingston Upon Hull, UK; 2 Imperial College London, UK

## Abstract

**Background:**

We present a case of redo aortic valve replacement in a patient with thrombocytopenia. The initial operation was performed through limited access transverse sternotomy. This is the first report of this kind in the literature.

**Case presentation:**

A 62 year old Caucasian male farmer with thrombocytopenia had uneventful redo aortic valve replacement when the first xenograft failed after 9 years, the transverse upper hemisternotomy in the first operation appearing to facilitate the redo complete sternotomy.

**Conclusion:**

With this only case of redo aortic valve replacement in our practice of 90 hemisternotomies over 10 years we present for consideration the use of a tissue valve in a complex relatively young patient.

## Background

**Figure 1:**
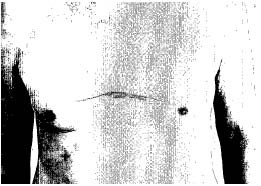
**Transverse sternotomy-minimal access option for access to pericardium: the skin incision**.

We present a case of redo aortic valve replacement with thrombocytopenia with a transverse hemisternotomy as the initial incision (Figure 1). Minimal access aortic surgery has been an established technique for over a decade [1].

## Case presentation

This 62 year old farmer with history of idiopathic thrombocytopenia (ITP) and rheumatic fever had required minimal access aortic valve replacement (AVR) with 23 mm Mitroflow Tissue valve for severe aortic stenosis and regurgitation in 1999: The xenograft was preferred over a mechanical prosthesis as he was unwilling to take warfarin for life. The first operation was performed through a transverse sternotomy at the level of the manubrio-sternal joint (Angle of Louis), the sternal flaps retracted cranio-caudally. Exposure was obtained to permit central aortic cannulation, venous cannulation and aortic valve replacement.

He was very well in himself until 2008, when he started having symptoms of exertional angina and occasional dizzy spells. Angiogram showed normal coronary arteries and Echocardiogram showed him to have a gradient of 50 across the aortic valve and some paravalvular leak with normal mitral valve. He again opted for a new tissue valve for similar reasons. His preoperative platelet count was 86,000/mm3; no preoperative therapeutic interventions were commenced in attempt to increase his platelet count.

On October 6, 2008, the patient underwent AVR with a 23 mm Carpentier Edwards pericardial valve through a redo median sternotomy. Access through the initial transverse hemisternotomy scar was not considered because of risks of considerable cardiac trauma in the presence of blood dyscrasia.

The adhesions were limited to the superior aspect of the anterior mediastinum and thence we found the exposure relatively facile. The prosthetic valve was found to have two torn leaflets with no paravalvular leak. The cardiopulmonary bypass (CPB) time under a standard perfusion protocol was 51 minutes and the aortic cross-clamp time 39 minutes. After neutralization of heparin with 200 mg Protamine, 2 units of platelets where pre-emptively transfused in view of ITP.

The patient was transferred to ICU in good haemodynamic condition with no bleeding initially. Two hours postoperatively, the total chest drainage being 1,130 mL, the patient was transfused 2 units of RBC, 4 units of FFP and 2 units of platelets. Total 24 hour drainage through the 3 chest wall drains was 2,430 mL (1200, 410,820) in diminishing hourly increments. The patient was transferred with the pericardial 32 French drain in situ to the low dependency area for convalescence and following repeat platelet counts was administered 75 mg of aspirin daily for antithrombotic modulation. The platelet count was 167,000/mm3 on post operative day 4 and 255, 000/mm3 on post operative day 8 and the patient was discharged.

## Discussion

Bleeding after redo cardiac surgery is a common occurrence with patients requiring re-operation to control bleeding as this may have disastrous haemodynamic effects. Pre-operative thrombocytopenia could theoretically increase the bleeding risk. With cardiac surgery requiring heparin for CPB adding an increased risk of reducing the platelet count and further increasing the risk of bleeding, the operation needs a specific decision pathway for patients with coagulation abnormalities.

The literature of cardiac surgery in ITP patients is scarce. ITP is primarily a disease of increased peripheral platelet destruction, with most patients having antibodies to specific platelet membrane glycoproteins. Preoperative high dose Immunoglobulin therapy for 4–7 days, steroid therapy and splenectomy have been practiced in ITP patients undergoing cardiac surgery.

In our patient, AVR was able to be carried out at a redo operation by means of intraoperative platelet transfusion post cardiopulmonary bypass and post operatively. Although the patient required blood transfusion few hours postoperatively the platelet count was not reduced (116,000 mm3) which could probably be related to reduced platelet function, postoperative blood transfusion was sufficient to control the bleeding.

Tissue valve is preferred over mechanical valve in patients with ITP as prosthetic valves may carry higher risk of lowering the platelet counts secondary to mechanical shearing of the platelets and also require lifelong warfarin. This may complicate the control of thrombocytopenia. Such patients are best managed with a haematologist with a view to appropriate platelet transfusion especially after weaning off the cardiopulmonary bypass to avoid significant bleeding after surgery.

## Conclusion

With this only case of redo aortic valve replacement in our practice of 90 hemisternotomies over 10 years we present for consideration the use of a tissue valve in a complex relatively young patient

## Competing interests

The authors declare that they have no competing interests.

## Authors' contributions

FH and ADP drafted the manuscript. SG co-authored the manuscript. All authors read and approved the final manuscript.

## Consent

Written informed consent was obtained from the patient for publication of this case report and accompanying images. A copy of the written consent is available for review by the Editor-in-Chief of this journal.
